# Hot topics and frontier evolution of growth-mindset research: a bibliometric analysis using CiteSpace

**DOI:** 10.3389/fpsyg.2024.1349820

**Published:** 2024-08-30

**Authors:** Jianmei Xu, Wenqiong Xu

**Affiliations:** ^1^Chinese Education Modernization Research Institute of Hangzhou Normal University, Hangzhou, China; ^2^Jinghengyi School of Education, Hangzhou Normal University, Hangzhou, China

**Keywords:** growth mindset, bibliometric analysis, CiteSpace, implicit theory, intervention

## Abstract

By virtue of CiteSpace, this study aims to evaluate and pinpoint the status, hot areas, and frontiers of growth-mindset research. Co-authorship analysis, co-citation analysis, co-occurrence analysis, cluster analysis, and content analysis are conducted, based on 543 articles selected from the Social Sciences Citation Index database. Researchers from Australia and countries/territories in North America, East Asia, and Western Europe have maintained relatively closer cooperation with each other. Carol S. Dweck, Jeni L. Burnette, David S. Yeager, and Mary Murphy have high publication volumes and close connections with each other. Angela Duckworth has acted as a bridge among many researchers. Highly co-cited literature has mainly focused on the impacts of mindset and intervention measures. In the past two decades, the literature on mindset research has plunged into numerous hot topics in terms of implicit theory, intelligence, motivation, beliefs, achievements, academic performance, students, transitions, and psychological intervention. Based on burst detection, the field of growth-mindset research shows the following trends: (1) future research must pay more attention to fidelity in intervention studies, conduct rigorous manipulation tests at the statistical level, and improve causal relationship models between growth mindset and other variables and (2) use a multidisciplinary perspective to provide a deeper explanation of the formation mechanism of the growth mindset. Finally, (3) the function mechanisms of the growth mindset in different cultural backgrounds should be strengthened.

## 1 Introduction

Being faced with the same problem, different individuals often have different attitudes and responses. One of the reasons for this underlies in the fact that people possess different mindsets. The study of mindset can matter-of-factly help us understand the world and thus transform it better (Reber, 1967). The origin of research on mindset could be traced back to the 1960s, with the boom in cognitive psychology and cognitive learning theory. This very theory, regarding the human as the subject of the learning process, posits that their behavior is the result of the interaction with environment. With this theoretical tide, Reber blazes a trail on implicit learning, and, soon after, Carol Dweck profounds the “implicit theories,” which is renamed after “mindset,” a belief or perspective down to the human traits (Dweck et al., 1995). The mindset theories, however, derive from the motivation theory, attribution and achievement motivation theory in particular (Dweck and Leggett, 1988).

The growth mindset, one of the two major types that Dweck first defined, is particularly tied to the *implicit theories of intelligence*. Those “theories,” according to Dweck, “were potentially falsifiable ideas about what intelligence is and how it might work,” and consists in the parallel of *entity* and *incremental theories*, while they are translated into “more user-friendly terms”—*fixed mindset* and *growth mindset* (Dweck and Yeager, 2019). The individuals with the former one are of the belief that their abilities are fixed into an “entity,” and thus they are failure-afraid and eager to flaunt their abilities, if were not gain them effortlessly. However, the individuals with *growth mindset* deem the “growth of abilities,” that is, their intelligence is expandable, malleable, and controllable and can be improved continually through effort, learning, and training. Moreover, they view the failures and setbacks more positively, and thus mostly achieve more in academic, professional fields or otherwise (Dweck, 2006). Not tough to conclude that, the study on *growth mindset* is an exploration of the personal factors in terms of emotion, will and interest, though they are easily overlooked, that work on the problem-solving *per se*. Therefore, to gain a panorama of the research status and envision the developing trends of *growth mindset*, this paper will take a systemic analysis on the literature of *growth mindset* collected from internationally important knowledge databases by means of bibliometrics and content analysis.

## 2 Data source and processing

To ensure the credibility and persuasiveness of the collected data, we selected literature for analysis from the Social Sciences Citation Index database on the “Web of Science” (WoS; Clarivate Analytics, London, UK). In conducting the CiteSpace bibliometric analyses, Web of Science, as the foremost comprehensive academic database globally, is renowned for its high-quality data records, detailed citation information, and rigorous screening criteria. We choose WoS to guarantee the elevated scholarly standard and dependability of the literature it encompasses, as this is essential for precisely establishing citation networks and pinpointing research frontiers and hotspots. Moreover, CiteSpace is particularly suitable for processing data from well-structured databases like WoS. It can effectively extract and analyze metadata like authors, institutions, keywords, and citation links, and subsequently produce visually intuitive analysis findings. Considering the purpose of the study and the functional suitability of CiteSpace, WoS became our preferred data source. The specific search strategy deployed in the SSCI database on the WoS was as follows: the topic search term was set as “growth mindset” with the search formula TS = (growth mindset), resulting in 1578 articles. Further refinement was done by selecting specific research areas, such as education, psychology, and social sciences, while excluding conference papers, editorials, book chapters. This yielded a total of 571 articles. To improve the accuracy and precision of the data, manual screening was conducted, resulting in a final selection of 543 articles. Changing the topic search term to “implicit theory,” the database yielded a limited number of articles, most of which were published a long time ago. It is worth noting that the majority of articles on implicit theories do not focus on intelligence or cognition. The *implicit theories of intelligence* are frequently utilized alongside development mentality, rather than separately, as precursor theories to *growth mindset*. Hence, the 543 articles acquired from the search phrase “growth mindset” are still utilized for subsequent research. Using the scientific literature measurement method and leveraging CiteSpace 6.2.R4 (Chaomei Chen, Drexel University, Philadelphia, PA, USA), an analysis was conducted on the current research status of the growth mindset. After removing duplicates, the total number of relevant articles was 543. The selected time span was from 2008 to 2022, with a time slice of 1 year. This analysis generated multiple types of visual representations, including a research hotspot visualization, a co-citation network visualization, and an emerging node visualization, to explore the current state and development trends of growth mindset research.

## 3 Numbers of publications

**Figure 1:**
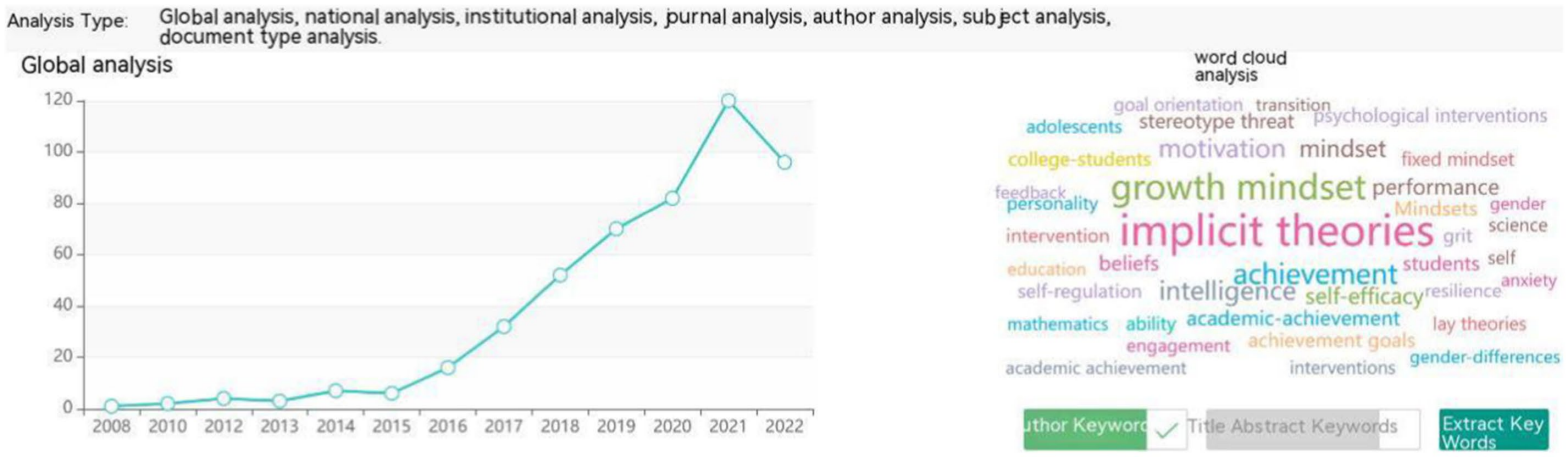
The number of “growth mindset” publications indexed in the WoS per year (2008–2022).

Using the Strategic Consulting Intelligence Support System from the China Knowledge Center for Engineering Sciences and Technology, the exported 543 foreign language articles were subjected to analysis. Figure 1 shows the yearly distribution of publications on “growth mindset” from 2008 to 2020. The research on “growth mindset” in fields such as education and psychology began in 2008. Initially, there was relatively low attention given to this field, and research remained in a nascent stage, with annual publication volumes of less than 10 papers before 2015. However, starting from 2015, there was a noticeable increase in publication volume, and publication counts continued to grow rapidly over the following 6 years, reaching a peak in 2021 with 120 articles. In 2022, there was a slight decrease in the annual publication volume, but the overall publication count remained stable at 96 articles. Overall, research on the growth mindset started early, experienced a flourishing period in the past 6 or 7 years, and has garnered sustained attention and interest.

## 4 Results

### 4.1 Core research forces

#### 4.1.1 Key researchers and Core author group

**Figure 2:**
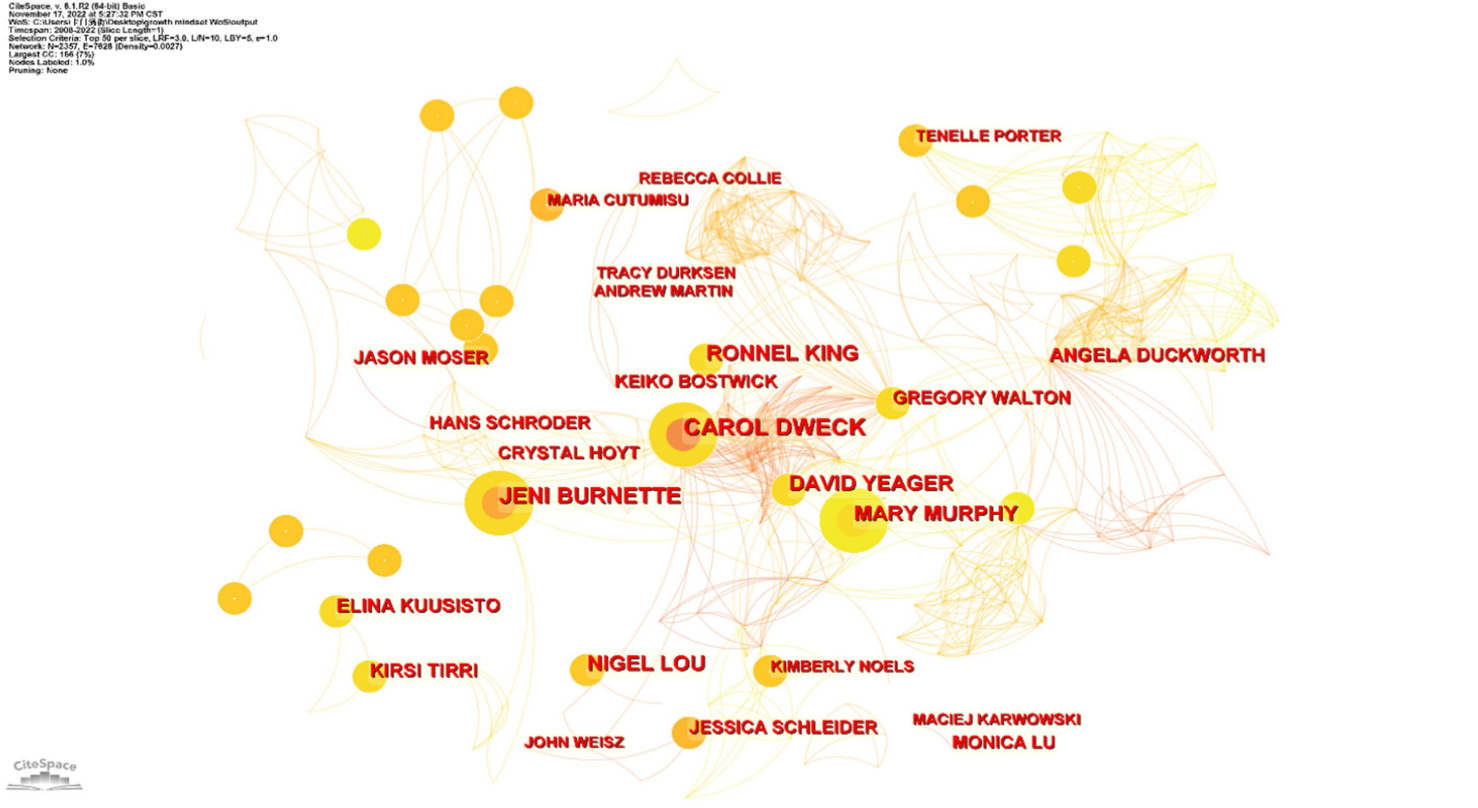
Core authors and their inter-relationships in the study of the growth mindset.

Using CiteSpace 6.2.R4, an analysis of authors and their inter-relationships among the selected literature was conducted. The specific steps involved selecting “Author” as the analysis node in the software’s parameter panel, choosing the top 50 authors based on their appearance frequency, and using the default “Cosine” calculation method for uncovering the connections. After adjusting the colors and dragging the nodes for clarity, an original visualization was developed and is shown in Figure 2.

The publications of core authors reflect the breadth and depth of research in the field. According to Price’s law, which determines the distribution of core authors, the relevant mathematical formula is as follows: M = 0.74√P_max_, where M represents the minimum publications of core authors during the statistical period and P_max_ represents the maximum publications of core authors during the statistical period. Based on our analysis, the P_max_ value for research on the growth mindset is 14 articles, so M was rounded to three articles. Therefore, authors with more than three publications are considered core author candidates. A total of 103 candidates were identified. These 103 authors have a combined publication count of 431, which exceeds half of the total publication count (543). This indicates the formation of a core author group in this field. As shown in Figure 2, the node size, node color, node connections, and line width represent the quantity of publications, the publication time, collaborative relationships, and the strength of relationships between authors, respectively. It can be observed that authors such as Carol S. Dweck, Jeni L. Burnette, David S. Yeager, and Mary Murphy have high publication volumes and close connections with each other.

Examining the node details of the aforementioned authors, it can be seen that Carol S. Dweck is the most productive author, with two first-authored and 12 co-authored articles indexed in the WoS Core Collection. Dweck is closely connected with high-productivity authors like Burnette and Yeager. According to our findings, Dweck has long been interested in the field of socio-cognitive development, particularly the relationship between students’ beliefs, motivation, and academic achievement. As early as 1993, she began collaborating with scholars Hong and Chiu from the Chinese University of Hong Kong to conduct research on implicit theories of intelligence, publishing several studies on cognitive influences based on this theory over the next 5 years (Dweck et al., 1993). These authors identify two different tendencies in cognitive processes: entity theory and incremental theory. Individuals with an entity theory tend to believe that personality is fixed, while those with an incremental theory believe that personality is malleable. This judgment continues to influence the problem-solving strategies adopted thereafter. Dweck believes that the likelihood of students’ academic success is influenced not only by their actual abilities but also by the goals and beliefs they hold in achievement situations. These beliefs and goals that influence individual behavior are not isolated but can be integrated into a meaning system, with the mindset integrating these variables to form this system (Dweck and Yeager, 2019). These studies have provided a solid theoretical foundation for her concept of the growth mindset. Based on our findings, Dweck could be considered a foundational and pioneering figure in research on the growth mindset.

By calculating the authors’ betweenness centrality, it is found that Duckworth has the highest betweenness centrality score of 0.01, playing a bridging role among many researchers and existing adjacent to several central nodes. By examining Duckworth’s articles indexed in the WoS Core Collection, it is observed that she has collaborated with core authors such as Yeager and Dweck in various areas, including interventions related to the growth mindset, assessment of non-cognitive skills (such as the growth mindset), and self-control. Duckworth has extensively studied grit, which is also a non-cognitive skill related to mindset, with the aim of predicting how it influences students’ academic and career achievements. She believes that the most effective way to cultivate grit is through the growth mindset (Duckworth, 2013).

#### 4.1.2 Countries (regions) and institutions of publications

**Figure 3:**
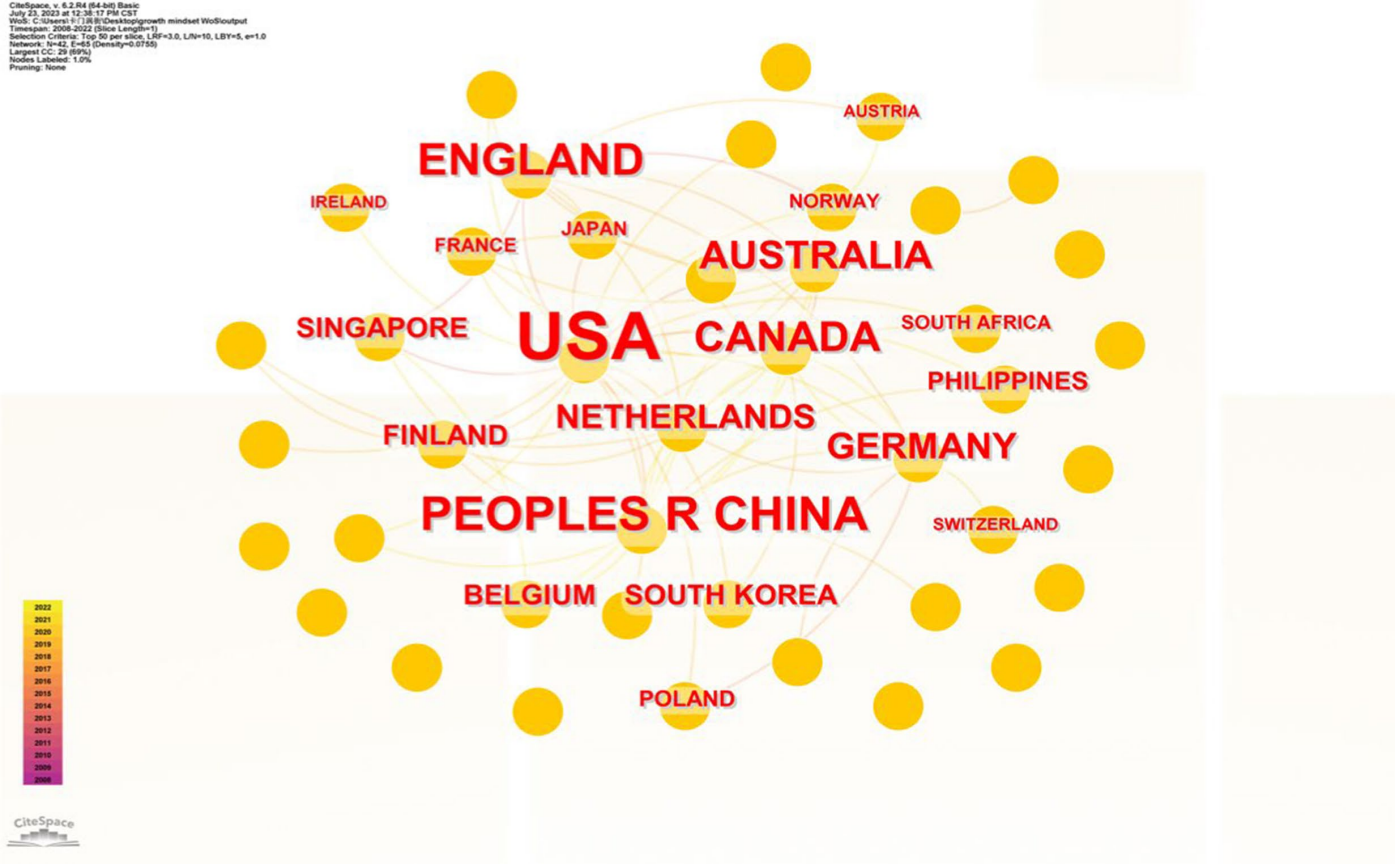
Distribution of authors by country (region) in the study of the growth mindset.

Using CiteSpace 6.2.R4, a further examination of the source countries and institutions of the selected literature is conducted to understand the overall situation. The resulting knowledge network is shown in Figure 3. In terms of the overall distribution, the literature collected in this study comes from 49 countries and regions. However, the top five countries (regions) in terms of publications account for a cumulative total of 478 articles, which is 88% of the total. North American countries are the main knowledge hubs, with the United States presenting as the central axis node radiating to the surrounding areas. East Asian countries, represented by China, Japan, and South Korea, form a secondary axis along with Western Europe and Australia. There are close cross-regional collaboration relationships among countries and institutions, and a collaborative network has basically formed.

**Figure 4:**
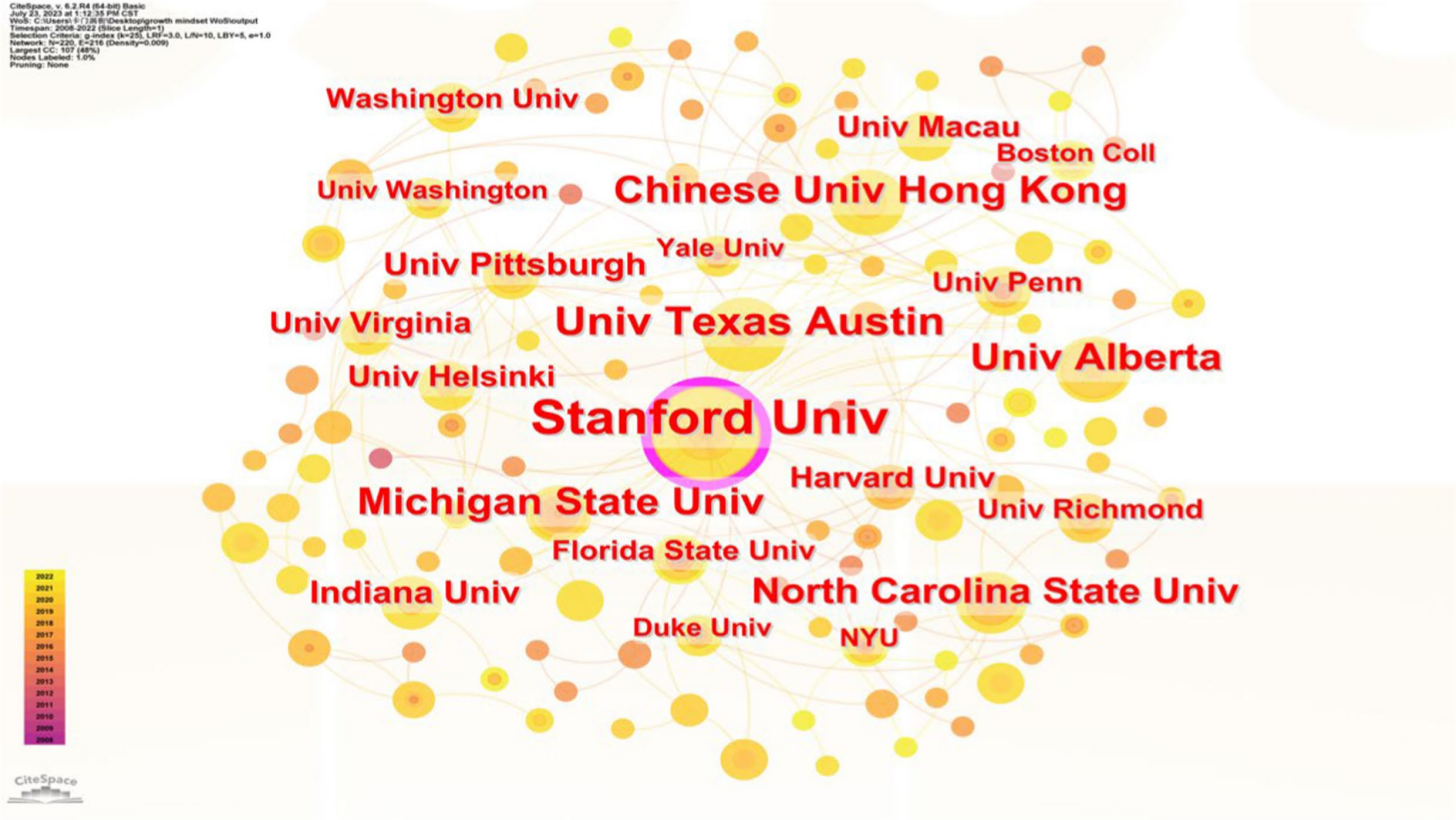
Institutional distribution of authors in the study of the growth mindset.

The country with the highest number of relevant publications is the United States. American scholars were the first to focus on and contribute to research on the growth mindset. Currently, 301 articles from the United States are indexed in the WoS Core Collection, accounting for a high proportion of 55%. Similarly, analyzing the distribution of institutions in the literature reveals that the majority of institutions are concentrated in certain universities in the United States. Stanford University, the University of Texas at Austin, Michigan State University, the University of Virginia, the University of Southern California, and Indiana University are particularly prominent as centers focused on researching the growth mindset (as shown in Figure 4).

China ranks second among all countries with a publication volume of 65 articles, but there is still a significant gap in publication counts compared to the United States. Growth mindset research in China was initially conducted by the University of Hong Kong, and, so far, 16 related articles have been indexed in the WoS Core Collection. The main research institutions in this area include the University of Hong Kong, the University of Macau, and Peking University. The University of Hong Kong has continued its tradition of researching the growth mindset due to its early collaboration with Stanford University on implicit theories. Notable scholars in this field include Chi Yue Chiu and Ying Yi Hong. The United Kingdom, Canada, and Australia follow China closely with publication volumes of 49, 34, and 29 articles, respectively. Major research institutions include the University of Cambridge in the United Kingdom, the University of Alberta in Canada, and the University of New South Wales in Australia, respectively.

### 4.2 Analysis of knowledge base based on spatiotemporal dimensions

#### 4.2.1 Spatial dimension

The top 24 authors with a publication volume of at least five articles were analyzed (refer to Table 1). Looking at the countries and research institutions to which these core authors belong, 13 scholars are from American universities, four are from Australian universities, three are from Canadian universities, and the remaining authors come from Finland (*n* = 2), Poland (*n* = 1), and China (*n* = 1). It can be observed that, except for the Chinese authors, the rest of the authors are from occidental countries.

**Table 1:** Major authors in growth mindset research.

Author	Country	Number of publications	Research field	Institution
Carol S. Dweck	USA	14	The Origins of Self-Concepts People Use to Structure the Self and Guide Their Behavior; Their Role in Motivation and Self-Regulation; and Their Impact on Achievement and Interpersonal Processes.	Stanford University
Jeni L. Burnette	USA	11	How to Implement Growth Mindset Interventions in Ways that Foster Self-Regulation and Improves Health, and How to Help Organizations Develop Growth Mindset Cultures.	North Carolina State University
Mary Murphy	USA	10	Self and Social Identity Threat; Stereotyping and Prejudice; Interracial Interaction and Friendship; Organizational Lay Theories; Structural and Psychological Barriers for Underrepresented Groups (women in STEM, students of color in academia)	Indiana University
Ronnel B. King	China	10	The Factors that Underpin Motivation; Well-being, and Socio-emotional Learning in K-12 and Higher Education Contexts; and Enhancing These Optimal Psychological States Through Leveraging on Positive Psychology/Education Interventions.	The University of Hong Kong
Nigel Lou	Canada	10	Beliefs and Motivation That Influence Language Development and Interpersonal Processes; Acculturation and Socio-Cultural Adaptation, Particularly Their Relations with Discrimination, Identity, and Language Development.	University of Victoria
David S. Yeager	USA	10	Social-Cognitive Development During Adolescence; Motivation; Behavior Change; Aggression and Bullying; Research Methodology and Psychological Measurement; Psychological Interventions.	The University of Texas at Austin
Kirsi Tirri	Finland	7	School Pedagogy; Moral and Religious Education; Talent Development and Gifted Education; Teacher Education and Cross-Cultural Studies.	University of Helsinki
Angela Duckworth	USA	7	Developmental Psychology; Individual Differences; Positive Psychology; Behavior Change.	University of Pennsylvania
Elina Kuusisto	Finland	7	Civic Purpose and Purposeful Teaching Among Students and Teachers; Ethical Sensitivity and Moral Education in Schools; Talent Development and Growth Mindset Pedagogy.	University of Helsinki
Hans Schroder	USA	6	Beliefs About Depression and Anxiety; Beliefs About Treatments for Mental Health; Public Health Messaging About Mental Health Problems.	University of Michigan
Crystal Hoyt	USA	6	The Role of Beliefs, Like Mindsets, Self-Efficacy, Stereotypes, and Political Ideologies, in the Experiences and the Perceptions of Individuals.	University of Richmond
Monica Lu	USA	6	How Dialogue and Social Interactions in the Classroom Facilitate Children’s Learning.	The Ohio State University
Jessica Schleider	USA	6	Developing Scalable, Brief Interventions for Youth Mental Health Problems, with a Focus on Depression and Anxiety; Identifying the Mechanisms of Change Underlying Their Effects.	Stony Brook University
Keiko C. P. Bostwick	Australia	6	Student Motivation; Teacher and Classroom Effects; and Quantitative Research Methods	University of New South Wales
Jason Moser	USA	6	Shedding Light on the Underlying Mechanisms of the Ability to Regulate Cognition, Emotion, and Behavior; Examining their Clinical Significance in Terms of Their Roles in the Development, Maintenance, and Treatment of Anxiety and Depression.	Michigan State University
Gregory M. Walton	USA	6	Self and Identity; Stereotypes; Motivation and Achievement; Psychological Intervention; Social Cognition.	Stanford University
Rebecca J. Collie	Australia	5	Examining Predictors and Outcomes of Different Factors Like Motivation, Wellbeing, and Social–Emotional Development Among Children, Youth, and Adults in Educational Settings.	University of New South Wales
Maria Cutumisu	Canada	5	Game-Based Assessments that Support Learning and Performance-Based Learning; Computational Thinking; AI in Games, and Virtual Character Behaviors in Interactive Computer Games with Applications in Education.	University of Alberta
Maciej Karwowski	Poland	5	Educational Assessment; Educational Leadership and Special Education.	University of Wroclaw
Tenell Porter	USA	5	Promoting Intellectual Humility in the Classroom; Cultivating Perseverance and Grit; Cultivating and Assessing Growth Mindset in Classroom Teaching.	Ball State University
Kimberly Noels	Canada	5	The Promotion Mechanism that LSocial Background and Intrinsic Motivation in the Process of Cross-Cultural Language Learning.	University of Alberta
Tracy Durksen	Australia	5	Teacher Education Research based on Social Cognitive and Self-Determination Theories.	University of New South Wales
John R. Weisz	USA	5	Mental Health Intervention Effects with Children and Adolescents Psychotherapy; Meta-Analysis.	Harvard University
Andrew Martin	Australia	5	Motivation, Engagement, Achievement, and Quantitative Research Methods; Research on Growth Mindset in the Field of Mathematics.	University of New South Wales

Combining information from Table 1 with the details of the core author group in the field of growth mindset research, along with their countries/regions, institutions, and research directions, we can analyze the foundation of the knowledge network constituting the growth mindset paradigm.

First, the research landscape presented by the knowledge map aligns closely with the institutions of the core authors in Table 1. Current research on the growth mindset is primarily centered around these core authors and carried out within their affiliated institutions. Authors from the same institution or country/region often have interconnected research interests. For example, core authors from the United States have primarily focused on adolescents, examining the development of their academic achievements and psychological well-being. Core authors from Australia have consistently directed their attention toward the field of teacher education, aiming to explore the impact of the growth mindset on teacher professional development and classroom instruction. Additionally, they have also emphasized the influence of the growth mindset on the domain of mathematics, including the relationship between academic achievement, engagement, and growth goals. Canadian scholars have demonstrated a strong interest in cross-cultural comparisons of the growth mindset and the mechanisms by which the growth mindset promotes language learning, particularly second language acquisition.

Secondly, there are close connections among the core author group, with frequent exchanges between academic institutions, and the existing collaborative networks tend to be biased towards core cities and major universities worldwide. Apart from collaborations within the same institution, there are also cross-institutional collaborations; for instance, Dweck and Yeager from Stanford University collaborated to explore the promotion of psychological resilience by the growth mindset (Yeager and Dweck, 2012), design intervention models reconstructing the growth mindset (Yeager et al., 2016a,b), and conduct large-scale online interventions to improve the educational trajectories of adolescents (Yeager et al., 2019). Porter from Pennsylvania State University concurrently lectures at Duckworth’s Grit Lab, collaborating on research related to fostering perseverance in adolescents through the growth mindset. The disciplinary backgrounds and research conditions of publishing institutions determine the quality and quantity of their affiliated literature. The research landscape mentioned above consists of renowned universities in various regions, which are located in economically developed areas and can provide excellent resources to support research.

In summary, the foundation of the knowledge network constituting the growth mindset paradigm is characterized by close alignment between the research landscape and the core authors’ affiliated institutions. The core authors often have interconnected research interests within the same institution or country/region while also maintaining strong connections and collaborations across institutions. These research institutions are well-known universities located in economically developed regions, which can provide favorable support and resources for research in the field of growth mindset research.

Furthermore, at the current stage, research efforts in different regions are primarily focused on intervention studies related to the growth mindset. Growth mindset interventions are typically considered as light touch interventions (Kim et al., 2022) and are common interventions that address both educational and mental health needs (Mosanya, 2021). However, investigators from different countries are approaching research from different angles, such as developing interventions based on neuroscience research, using computer games for intervention therapy, and developing single-session online intervention models, among others. Looking at the development process of mindset research, we can see that each stage has its own characteristics.

In the first stage of research, the focus was mainly on demonstrating the positive impact of the growth mindset on individual intelligence and academic development, such as its promotional and predictive effects on seeking challenges, positive outcomes, and psychological resilience. Additionally, mindset also influences the formation of social judgments and biases in interpersonal interactions. In the second stage, which is the current stage, research on mindset has entered the era of field experiments and replication science. Its characteristic is the use of large samples and longitudinal designs to examine changes in mindset processes (Dweck and Yeager, 2019). Unlike in previous laboratory experiments, the current challenges posed by field experiments and the difficulties in developing intervention measures have become common concerns among scholars worldwide.

Lastly, in the context of accelerating globalization, research on the growth mindset should not be limited to a single country or region. Researchers have started to adopt a comparative perspective to examine the development of a growth mindset and focus on the similarities and differences in the growth mindset across different cultural backgrounds. Moreover, the participation of researchers from different socio-cultural backgrounds has allowed research team expansion; broadened the research perspective; and even led to the establishment of closely connected and productive scholar networks like the Student Experience Research Network (SERN), which conducts research on the growth mindset and stereotype threats. SERN scholars come from 24 different institutions and cover various stages of development from early childhood to adulthood. Although the network operations have reached a conclusion in 2023, this interdisciplinary growth mindset approach to research continues to exert an influence on future research, and the network’s resources remain accessible.

#### 4.2.2 Time dimension

**Figure 5:**
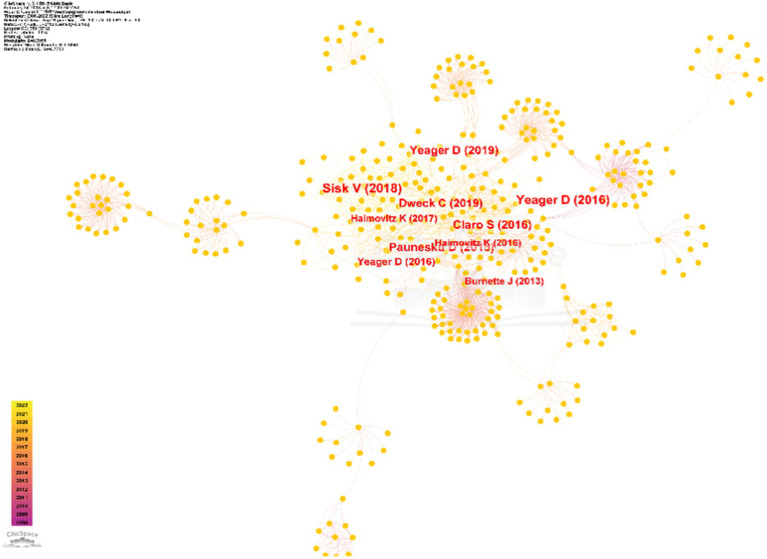
Distribution of highly co-cited literature in the study of the growth mindset.

The knowledge foundation can reflect the essence of cutting-edge research in a field, with highly cited literature best representing the knowledge foundation of that research area. By conducting co-citation analysis of the collected literature’s references, as shown in Figure 5, it was revealed that the size and color depth of the nodes are positively correlated with their citation frequency.

By filtering out highly co-cited literature with a citation frequency of at least 30 citations, we identified 10 highly co-cited papers (Table 2). Among the authors involved in these papers, Yeager has published three papers. The citations of these papers mainly occurred from 2016 to 2019, with a concentration in 2016. However, there have been no highly cited papers in the past 2 years. The network graph exhibits a clustering distribution pattern characterized by “multi-core” and “periphery” nodes. There are strong cores formed by highly co-cited papers closely connected to each other as well as multiple peripheral clusters radiating from single weak core nodes. When combining this information with the results of the analysis of the core research forces mentioned earlier, we could discern that the prominent nodes in the co-citation network align well with the core author group presented in the previous discussion.

**Table 2:** Highly cited literature on growth mindset research (frequency ≥ 30).

S. No.	Author	Citation frequency	Publication year	Highly co-cited articles
1	Victoria F. Sisk	93	2018	To What Extent and Under Which Circumstances Are Growth Mind-Sets Important to Academic Achievement? Two Meta-Analyses
2	David S. Yeager	90	2016	Using Design Thinking to Improve Psychological Interventions: The Case of the Growth Mindset During the Transition to High School
3	David S. Yeager	71	2019	A National Experiment Reveals Where a Growth Mindset Improves Achievement
4	Susana Claro	70	2016	Growth Mindset Tempers the Effects of Poverty on Academic Achievement
5	David Paunesku	69	2015	Mind-Set Interventions Are a Scalable Treatment for Academic Underachievement
6	Carol S. Dweck	51	2019	Mindsets: A View From Two Eras
7	David S. Yeager	39	2016	Teaching a Lay Theory Before College Narrows Achievement Gaps at Scale
8	Kyla Haimovitz	32	2017	The Origins of Children’s Growth and Fixed Mindsets: New Research and a New Proposal
9	Kyla Haimovitz	32	2016	What Predicts Children’s Fixed and Growth Intelligence Mind-Sets? Not Their Parents’ Views of Intelligence but Their Parents’ Views of Failure
10	Jeni L. Burnette	30	2013	Mind-Sets Matter: A Meta-Analytic Review of Implicit Theories and Self-Regulation

Tracing the development of highly co-cited literature based on publication years provides insights into the research trajectory of a field. The attention to mindset began shifting from exploring the mechanisms of mindset to focusing on intervention measures and replication in the 2010s. The interventions based on a growth mindset showed promising prospects for improving students’ academic achievements in face-to-face interactions. However, the limitations of these “experiential interventions” in terms of time and cost have hindered their potential for widespread implementation and replication. Experimental data alone could no longer meet the current demands of large-scale educational improvement nor enhance educational equity, thus requiring a shift toward large-scale replication in intervention research (Paunesku et al., 2015). Over the past decade, researchers have made efforts to develop concise and practical online intervention programs, yielding substantial data. However, the data also suggest that there are positive and negative poles in the impact of online interventions on different groups of students, such as struggling students versus high-achieving students. Moreover, in real educational settings, the inability to strictly control and estimate the influence of covariates makes it challenging to ensure that interventions have the intended impact on participants rather than other mediator variables (Sisk et al., 2018). Easily implementable interventions may not necessarily be easy to develop or widely disseminate. Yeager improved intervention measures using design thinking principles, incorporating qualitative surveys to gather information on preferred intervention methods among the target population, such as storytelling using examples of famous individuals, peer role models, diversified writing exercises, and reducing information overload during the intervention. Randomized A/B testing was conducted on participants, followed by self-report evaluations. Meanwhile, the “saying-is-believing” approach helped participants internalize the growth mindset (Yeager et al., 2016a,b).

Interventions are not limited to classroom environments; they are also crucial during important transitional periods in individuals’ lives, such as school choice. Students often feel confused and uncertain during such transitions. Academic struggles often begin for students during early adolescence and can persist. Analyses revealed that changes in various aspects of the school environment, as perceived by students, particularly alterations in teacher support, could to some extent forecast changes in levels of student academic, personal, and interpersonal functioning (Barber and Olsen, 2004). Furthermore, a longitudinal study has confirmed that students who fall behind in middle school are at a higher risk of dropping out of high school (Bowers, 2010). Preparatory interventions based on a growth mindset can support students in developing higher strain levels in the future, enabling them to better adapt to changing environments. However, research suggests that the combined use of interventions targeting academic achievement and a growth mindset does not always yield greater effectiveness compared to their individual use, highlighting the importance of the delivery method for promoting growth mindsets (Yeager et al., 2016a,b).

Intervention measures are rapidly evolving in ways that are more easily accepted by groups. At the same time, intervention research, while penetrating deeper into schools, is also shifting its focus to the broader mechanisms influencing the growth mindset within society and families. Researchers, considering causal relationships derived from previous raw data, are employing quantitative research methods like meta-analysis to re-examine these relationships in larger sample sizes, aiming to explore the interplay of multiple factors. Socioeconomic conditions, as a structural factor, have been found to influence mindset mechanisms. Individuals with a stronger growth mindset can effectively mitigate the detrimental effects of low socioeconomic conditions on future achievement, and the positive effects of a growth mindset hold true across different socioeconomic strata (Claro et al., 2016). In addition to socioeconomic conditions, there is a growing body of evidence shows that teachers can create classroom cultures that are consistent (or inconsistent) with growth mindset. This can affect students’ perceptions of or reactions to the context (Muenks et al., 2020; LaCosse et al., 2021). A research based on a hypothesis proposes that the effects of individuals’ newly adopted beliefs depend on environmental affordances. The impact of these interventions may be amplified in settings where the pertinent beliefs and their associated behaviors are more malleable. Conversely, it is also plausible that a conducive environment could result in diminished estimated effects of growth mindset interventions. Building on this study, researchers have developed the Mindset × Context framework to understand this heterogeneity phenomenon that the different intervention effects for people in different contexts. This framework can interpret emerging evidence and guide the next generation of research on belief-supporting interventions, complementing established belief-changing interventions. The cues or features of the context permit or encourage individuals to internalize and act on their new mindsets. Concurrently, the results of previous large-scale, multi-site randomized trials (e.g., Yeager et al., 2019; Rege et al., 2021) indicate that there is a positive interaction between student interventions and contextual support. This implies that these interventions are more effective in more supportive environments. The full Mindset × Context framework integrates this positive interaction into a broader model that demonstrates how the effects of direct-to-student interventions could be modified by individual and contextual factors (Hecht et al., 2021). A large-scale, randomized controlled trial demonstrates that growth mindset interventions are more effective when delivered by teachers (Porter et al., 2022).

Furthermore, the family environment and parental parenting style also influence the development of children’s growth mindset. Non–mindset-related beliefs naturally permeate and influence children’s mindset formation. The influence of adults, such as parents and teachers, on children’s mindset formation often goes beyond their own mindset. Instead, their other beliefs, such as beliefs about failure and how they motivate children, manifest in visible attention and behaviors, shaping children’s beliefs in return. Children can accurately perceive parents’ beliefs about failure, which in turn predicts their beliefs about intelligence (Haimovitz and Dweck, 2016, 2017). This finding opens up directions for future research, focusing on the bidirectional influence between parents and children and the reciprocal interactions with other environmental factors. This aligns with Dweck and Yeager’s prediction regarding the research shift—namely, a greater focus on the mindset environment in which individuals are situated.

### 4.3 Analysis and evolution trends of research hotspots in growth mindset studies

By tracing the knowledge base of growth mindset research based on the temporal and spatial dimensions, the analysis of keyword co-occurrence and clustering in CiteSpace 6.2.R4 allows us to explore the interconnectedness of keywords in selected articles and infer the research foci in the related field.

#### 4.3.1 Analysis of research hotspots based on keywords

**Figure 6:**
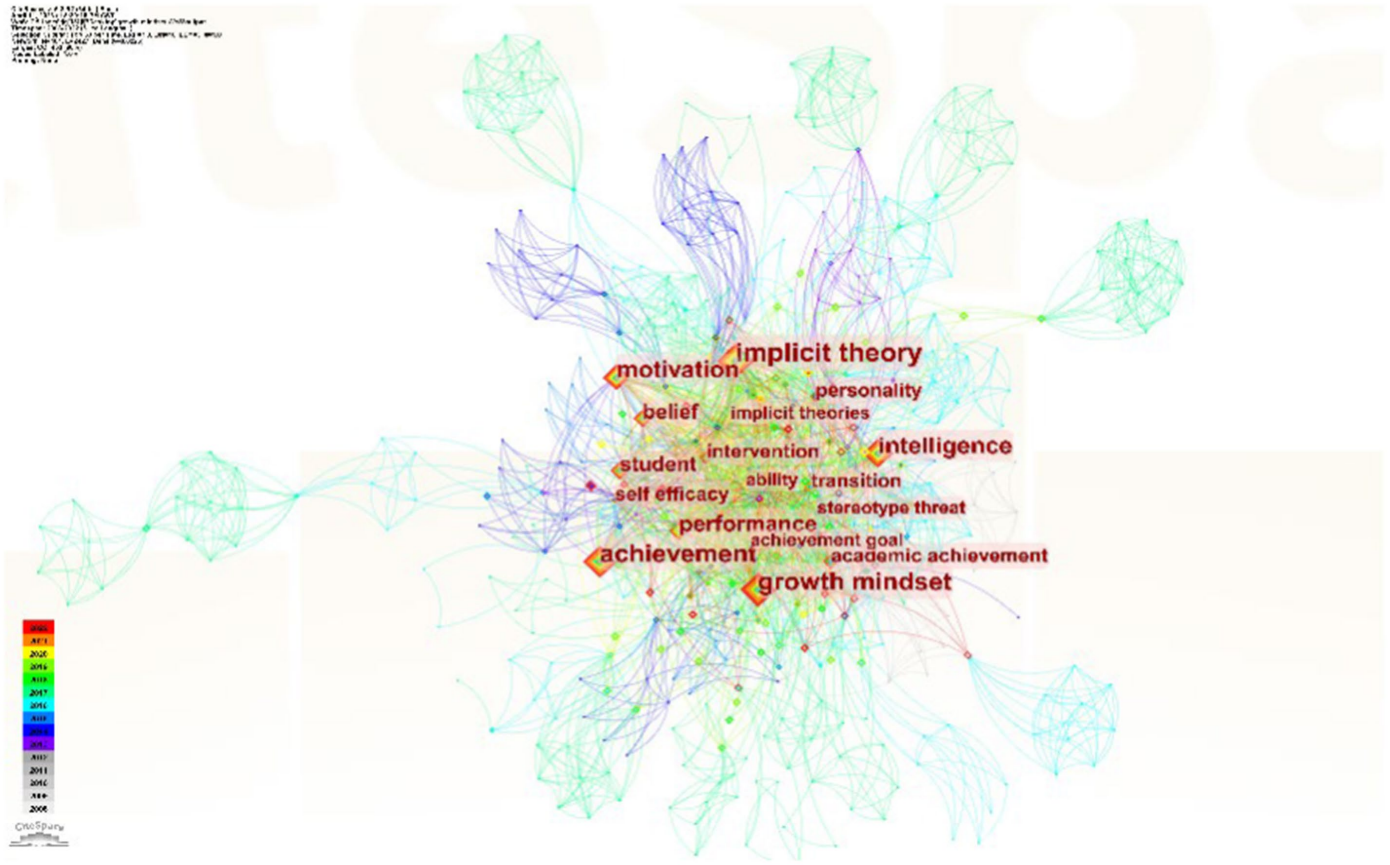
Literature keyword co-occurrence in the study of the growth mindset.

Keywords reflect the themes and central ideas of an article, summarizing its content. By using the “keyword co-occurrence” function in CiteSpace 6.2.R4, we analyzed the interconnectedness of keywords in the selected articles to identify research hotspots and their evolution trends. After visualizing the original network map, further modifications of specific parameters in the control panel are made, resulting in the exported Figure 6.

**Figure 7:**
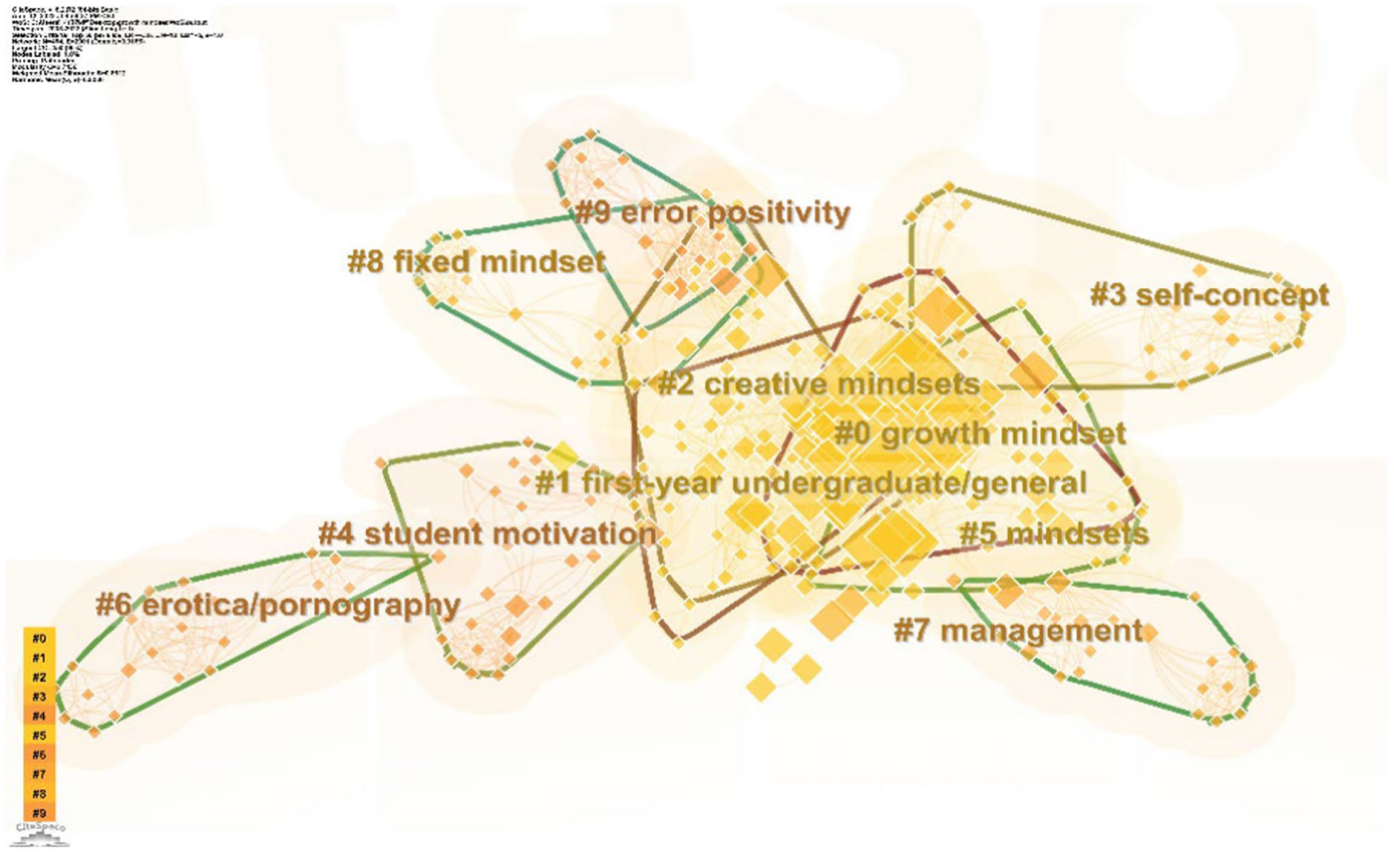
Literature keyword clusters in the study of the growth mindset.

Building upon the “keyword co-occurrence” analysis the “keyword clustering analysis” was performed yielding 21 clusters. The 10 largest clusters are selected and their silhouette profiles are presented in Figure 7. The Q-value (modularity Q) was 0.7436 and the S-value (weighted mean silhouette S) was 0.8812. Based on these values we concluded that the network structure was significant the clustering results were good and the credibility was high. The size of each silhouette represents the scale of the cluster with larger sizes indicating more related keywords within the cluster and a greater level of attention to that topic.

In Figure 7, we can observe that there is a partial overlap of cluster #0 with clusters #2 and #5. These clusters contain large and densely distributed keyword nodes, indicating the presence of numerous high-frequency keywords related to the theme of “mindset.” The topics covered include implicit theories, stereotypes, research subjects related to mindset, and studies on mindset interventions. Cluster #3 is related to “self-concept,” while cluster #4 is associated with “student motivation.” These clusters include keywords related to internal psychological aspects, such as “goal-setting” and “academic beliefs,” as well as keywords related to external factors, such as “Chinese parenting” and “psychological control.” Clusters #6 and #7 are relatively independent and primarily focus on interpersonal relationships. Alongside keywords like “management” and “leadership,” topics related to sexual relationships, such as “sexual satisfaction” and “erotica/pornography,” are also included. Clusters #8 and #9 also have some overlap. Cluster #8 revolves around the keyword “stress” and explores the relationship between mindset formation and individual cognition and behavioral experiences, including keywords like “fixed mindset” and “stress-related growth.” Cluster #9 approaches mindset formation from a neuroscientific perspective, focusing on brain electrophysiological signals related to error processing, such as “error positivity” and “error-related negativity,” to investigate the generation and impact of mindset.

Additionally, keywords related to research methods and processes, such as “meta-analysis,” “structural equation modeling,” and “reliability of measures,” are dispersed across various clusters, reflecting the emphasis on quantitative methods in growth mindset research. To provide a clearer presentation and analysis of the data, a “summary table” of the keyword data was exported, and Table 3 was obtained after consolidating singular and plural synonyms. This table summarizes the high-frequency keywords in the field of growth mindset research and, combined with the presented clusters, identifies four research hotspot directions.

**Table 3:** High-frequency keywords in the study of growth mindset (2008–2022).

Research hotspots		Key words	Frequency
Theoretical Origins	Precursor Theory	Implicit Theory	259
		Growth Mindset	228
		Intelligence	175
	Conceptual Definition	Motivation	140
		Belief	87
		Personality	54
		Attribution	32
		Grit	11
Influencing Factors	Influencing Factors	Self-efficacy	51
		Stereotype Threat	37
		Goal Achievement	35
		Self-regulation	20
	Effected Factors	Achievement, Academic Achievement	246
		Performance	114
		Ability	42
		Engagement	25
		Success	18
		Behavior	17
		Health	8
Research Subjects		Students	88
		Transition	57
		College Students	29
		Adolescents	18
		Higher Education	16
		Gender Differences	16
Research Direction	Research Approach	Intervention, Psychological Intervention	86
	Research Method	Model	29
		Meta-analysis	17

The theoretical origins of the growth mindset can be traced back to the concept of implicit theory, which is reflected in the highest co-occurring keyword “implicit theory.” Dweck proposed two theoretical frameworks of ability based on individuals’ different understandings of intelligence; these are the entity theory, which views intelligence as fixed, and the incremental theory, which sees intelligence as malleable. From these frameworks, the fixed and growth mindset concepts were developed (Dweck and Yeager, 2019). The scientific validation of the growth mindset theory extends beyond the psychological level to include physiological aspects. Myers et al. approached the topic from a neuroscience perspective; using functional magnetic resonance imaging techniques, they conducted a study on 20 children with an average age of 11.2 years and identified a correlation between brain regions involved in error monitoring, such as the dorsal anterior cingulate cortex, and the influence of the growth mindset (Myers et al., 2016). Current research utilizing brain activity measurement techniques such as electroencephalography is gradually uncovering the neuroscientific basis of the growth mindset. By reviewing the theoretical lineage of mindset research, we can see that the dominance of behaviorism in psychology gradually declined from the 1960s, leading to the cognitive revolution. Studies on learned helplessness in animals revealed the mechanisms by which experiences form beliefs and how beliefs influence motivation (Weiner and Kukla, 1970). Dweck and Yeager combined animal learning theory with the emerging theory of individual attribution, exploring whether individuals would interpret uncontrollable and unpredictable events differently and how these interpretations shape their behavior (Dweck and Yeager, 2019). Subsequent research confirmed this hypothesis, suggesting that children’s attributions for failure can predict their helplessness or mastery-oriented responses to setbacks (Dweck and Reppucci, 1973). Through a series of experiments, researchers raised the question of why, among students with relatively equal abilities, some are more concerned with proving their abilities, while others are more focused on improving their abilities. To address this question, the field of mindset research emerged.

## 5 Discussion

### 5.1 Exploration of factors influencing the growth mindset

With the flourishing development of online teaching and learning, which differs from traditional face-to-face formats, students now require higher levels of self-regulation skills for autonomous learning planning. Consequently, more research has focused on factors influencing the growth mindset from the individual’s intrinsic perspective, such as self-efficacy and self-regulation. Jeni Burnette and colleagues approached the topic from the theory of self-control, highlighting the direct relationship between an individual’s implicit theory of intelligence and self-regulatory processes like goal-setting and monitoring (Burnette et al., 2013). The external environment is also an important influencing factor, with investigators exploring how individuals use environmental cues to construct their mindset. When studying the impact of mindset formation, researchers often examine the effects of different environmental factors within educational contexts. These factors are evaluated based on individual academic achievements, such as course grades, standardized test scores, and GPA or participation and completion rates for advanced courses and challenging tasks (Yeager et al., 2019). Some scholars have shifted their focus to the overall individual growth process and pay more attention to psychological well-being. Lawrence Rescorla from Stanford University addressed the issue of adolescent drug and alcohol addiction, highlighting the irreversible effects on neurotransmitters and the continuous need for medical intervention. Based on these findings, Burnette and other researchers have attempted to change participants’ fixed mindset beliefs regarding the incurability of addiction by inducing growth mindset messages. However, the results showed minimal effects of the growth mindset on cessation treatment (Burnette et al., 2019).

Currently, more research simultaneously focuses on both of the aforementioned aspects of influence. One study based on data from the Programme for International Student Assessment examined the scores of students from different socioeconomic backgrounds in reading, mathematics, and science as well as their satisfaction with life and happiness indices. The goal was to determine the role of the growth mindset in bridging the gap between disadvantaged and high-achieving students. This study found that, on average in PISA, students who reported having a growth mindset scored higher in reading, mathematics, and science, displayed lower levels of fear of failure, and are more likely to consider their life satisfactory. Growth mindset is associated with a larger score gain for girls (+3 points), and disadvantaged (+12 points) and immigrant students (+9 points) when compared to boys, and advantaged and non-immigrant students (Gouëdard, 2021).

### 5.2 Diverse research subjects in the study of the growth mindset

As indicated by the previous information, research on mindset has primarily focused on surveying students, particularly those in higher education or the transitional phase of middle and high school during adolescence. Many studies targeting the transitional period in basic education confirm that adolescence is a critical period for students facing numerous academic and social challenges. Students may encounter various difficulties, such as a reduced sense of belonging due to changes in the school environment, declining academic performance due to increased difficulty, and increased participation in challenging courses. The flexible changes in the educational trajectory during secondary school often lead to neglect in identifying and effectively addressing these problems. Research indicates that, over time, the issues that manifest in early secondary school will compound into significant differences in human capital in adulthood (Yeager et al., 2019). Shifting to higher education, newly enrolled university students also receive considerable research attention. The transition in environment that occurs when entering college signifies that more complex challenges lie ahead. Moreover, increasing age does not necessarily mean enhanced stress resistance or a better ability to cope with challenges among college students. Judd specifically examined the significance of the growth mindset for learners in higher education environments. He believes that the impact of the growth mindset includes resilience, perseverance, persistence, social and teamwork skills, and giving and receiving peer support. These are essential general abilities for the comprehensive development that college students strive to acquire (Judd, 2017). Almost every empirical study has conducted gender comparisons, yet no significant gender differences have been observed in the mindset held by individuals.

### 5.3 Expansion of application areas and research methods in the study of the growth mindset

Intervention research is a key focus in the application of the growth mindset, having been extensively applied to academic achievements, social qualities, and psychological health interventions. Studies on growth mindset interventions span from primary education to higher education, covering multiple disciplines and fields such as language, mathematics, and entrepreneurship. A “pure” growth mindset does not exist; in other words, each individual is a mixture of fixed and growth mindsets, and this mixture evolves continuously with experience (Dweck, 2000). Adolescence is an important period for personality formation and the prevalence of psychological issues. It is also a golden period for interventions. Therefore, the majority of intervention measures are targeted at the adolescent stage. Burnette et al. conducted comparative experiments on different intervention models in an entrepreneurship introductory course and found that the growth mindset promotes entrepreneurial self-efficacy and simultaneously enhances academic and career interests among adolescents (Burnette et al., 2020). Even brief face-to-face interventions can change some traits related to adolescence and society; reduce aggressive behavior; and address the widespread issues of campus violence, racial discrimination, and fixed mindset stereotypes (Yeager et al., 2013). In terms of the development and implementation framework of intervention measures, Ku and Stager selected and examined 20 empirical studies on the effectiveness of growth mindset interventions; specifically, they extracted self-regulation, self-efficacy, and self-worth as three sub-abilities of the growth mindset and developed a more feasible intervention framework for higher education practitioners based on these concepts (Ku and Stager, 2022). Some investigators have pointed out that, when focusing on primary school, research in this area is often limited in scale and tends to confuse overall school process evaluations with targeted intervention measures (Savvides and Bond, 2021). Although growth mindset interventions are widely applied in classroom practices, this emerging field still requires more rigorous implementation procedures and outcome research.

### 5.4 Prediction of evolutionary trends based on emergent nodes

The concept of burst detection refers to a sharp increase in the citation frequency of a specific paper within a short period. This indicates that scholars have paid close attention to the content of that research field, reflecting the changes and dynamics of the research topics in the relevant field. By selecting “burst detection” on the control panel, the generated co-citation knowledge graph displays 25 high-bursting papers published from 2008 to 2022. In this analysis, we selected 11 papers with burst intensities of greater than 6 for further examination (Table 4).

**Table 4:** Articles with the strongest citation bursts (intensity ≥6).

S. No.	Author	Publication year	Strength	Timespan	Article
1	David S. Yeager	2019	21	2020–2022	A National Experiment Reveals Where a Growth Mindset Improves Achievement
2	David Paunesku	2015	17.43	2017–2020	Mind-Set Interventions Are a Scalable Treatment for Academic Underachievement
3	Victoria F. Sisk	2018	15.02	2019–2022	To What Extent and Under Which Circumstances Are Growth Mind-Sets Important to Academic Achievement? Two Meta-Analyses
4	Jeni L. Burnette	2013	13.09	2017–2018	Mind-Sets Matter: A Meta-Analytic Review of Implicit Theories and Self-Regulation
5	David S. Yeager	2016	11.88	2019–2022	Using Design Thinking to Improve Psychological Interventions: The Case of the Growth Mindset During the Transition to High School
6	Ana Costa	2018	9.58	2020–2022	Implicit Theories of Intelligence and Academic Achievement: A Meta-Analytic Review
7	Susana Claro	2016	8.63	2019–2022	Growth Mindset Tempers the Effects of Poverty on Academic Achievement
8	Carissa Romero	2014	7.43	2017–2019	Academic and Emotional Functioning in Middle School: The Role of Implicit Theories
9	Elizabeth A. Canning	2019	7.15	2020–2022	STEM Faculty who Believe Ability is Fixed Have Larger Racial Achievement Gaps and Inspire Less Student Motivation in their Classes
10	Daeun Park	2016	6.52	2019–2020	Young Children’s Motivational Frameworks and Math Achievement: Relation to Teacher-Reported Instructional Practices, but Not Teacher Theory of Intelligence
11	Štěpán Bahník	2017	6.24	2019–2020	Growth Mindset is Not Associated with Scholastic Aptitude in a Large Sample of University Applicants

Based on the analysis of the emergent node literature and the research directions of the main researchers in this field, as well as the four research hotspots identified earlier, we can make some predictions about the future evolution of research. For example, it is expected that the research procedures in the study of the growth mindset will be improved, including the measurement of data, data-collection methods, and statistical processes. The existing mature scales include Dweck’s Growth Mindset Scale developed in 2000, which has good reliability, validity, and cultural adaptability. As researchers delve deeper into the concept, more and more scholars are attempting to assess the level of growth mindset in research subjects from multiple attribute perspectives, such as beliefs about intelligence, effort, and setbacks. At the same time, many research errors and heterogeneity are related to the data-collection process. Future research needs to pay more attention to fidelity in intervention studies, conduct rigorous manipulation tests at the statistical level, and improve causal relationship models between the growth mindset and other variables.

Second, the applied attributes of the growth mindset will continue to be strengthened in contemporary research. After overcoming the limitations of small sample sizes in intervention studies, contemporary researchers are gradually conducting larger-scale assessments and interventions using remote online operations and big data computations. Large-scale testing often entails a large amount of data collection, and current studies often conduct interventions on a monthly or semester basis. While short-term measurements have shown promising significant effects, research data with longer timespans show that the effects of growth mindset interventions are comparable to traditional interventions, with low effect sizes. Some scholars have pointed out the existence of “publication bias” in the field, where the effect sizes of published studies are much larger than those of unpublished studies, or there is “*p*-value manipulation,” where research data are selectively reported (Paunesku et al., 2015). Small effects, null effects, or effects in non-predicted directions are often discarded by researchers, while influential conclusions are often based on a few inconspicuous heterogeneous samples. Future research may need to focus more on heterogeneous samples that emerge and investigate the reasons for heterogeneity and low effect sizes. In addition to procedural adjustments in quantitative research, qualitative research often involves self-reporting by participants to investigate their mindset, such as self-reporting in teacher classroom instruction. There may be response biases, such as social expectation errors, that can subjectively influence the results (Park et al., 2016). Therefore, simultaneously examining self-reporting and specific practices can greatly improve the objectivity of the research. Future research should pay more attention to details and subtle differences, such as handling heterogeneous samples, controlling research variables, reducing research subjectivity, and better integrating quantitative and qualitative methods to improve research procedures.

Furthermore, the exploration of the mechanisms underlying the formation of the growth mindset will deepen. Apart from the influence of physiological mechanisms, such as the neuroscientific basis of growth-mindset formation, there are complex and variable social environmental factors of interest. Park et al.’s research indicates that teacher performance–oriented teaching practices have a significant impact on the formation of students’ fixed mindsets. The data show a negative correlation between teacher performance–oriented teaching practices and students’ math scores over one academic year, although this finding is not significant at the statistical level. However, when the research period was extended to two academic years and measured continuously, the data became significant. This suggests that external influences on an individual’s growth mindset can accumulate over time. Going further back, it can be confirmed that belief differences in mindset and human attribute plasticity have already been identified in early elementary school children, but they are not domain-specific (Park et al., 2016). Research on the conditions for mindset formation also paves the way for exploring the function mechanisms in subsequent studies, with the goal of better leveraging the positive effects of the growth mindset.

Similarly, previous studies on the impact mechanisms of parenting styles on children’s mindset formation corroborate Park’s conclusion that the thinking environment in which children grow up in strongly influences the formation of their mindset. Yeager et al.’s research found that prior achievement levels and peer norms in the middle school transition period can influence mindset transitions. High-achieving students often receive more and higher-quality resources and are less likely to change, and positive peer norms facilitate the formation of the growth mindset in specific domains (e.g., reading; Yeager et al., 2019). The emphasis on the growth environment of children’s thinking also prompts researchers to pay more attention to the interactive effects between multiple mechanisms, such as simultaneously examining the interactive effects of parenting styles and school classroom environments. In addition to tracking measurements in real-life contexts, related laboratory studies can be conducted to explore causal relationships more accurately. Furthermore, an increasing number of studies have infused interdisciplinary perspectives, strengthening the foundation of explanatory mechanisms for the growth mindset.

Lastly, research on the function mechanisms of the growth mindset will become more precise. As the growth mindset is applied in a wider range of disciplines and fields, the issue of how to more accurately control complex variables to maximize the function of the growth mindset has become a common concern. Studies have shown that the growth mindset has a greater impact among students with a lower socioeconomic status and provides more help to them (Claro et al., 2016). In contrast, among high-achieving students, the scope of improvement is smaller and the impact of the growth mindset is weaker (Yeager et al., 2019). Moreover, scholars have focused on the involvement of minority groups in STEM classrooms and pointed out a negative correlation between teachers’ fixed mindset and minority students’ participation (Canning et al., 2019). These findings urge us to investigate the integration of mindset in different thinking environments, and relevant questions include the following: how can we best convey a growth mindset to different individuals? How does the organization of the thinking environment determine whether students accept and apply new thinking or help embed the growth mindset into the culture of schools and organizations? Finally, how should we address the potential adverse effects of mindset? We still know very little about the best answers to these questions. Romero et al. demonstrated the predictive role of mindset for students’ academic performance by measuring the scores of middle school students and the enrollment rate in AP math courses. They confirmed the predictive role of affective theory for students’ emotional functioning by tracking measurements of depression and well-being among middle school students. However, upon examining the interaction effects of these two mechanisms, it was found that the results were not significant (Romero et al., 2014). Similar to the generation mechanisms of mindset, the function mechanisms of mindset also involve the possibility of different theories and environmental interactions.

In addition, existing research on school learning and transitional periods has, to varying degrees, demonstrated the positive function of the growth mindset for different populations. Some scholars have pointed out that the influence of a growth mindset only manifests in adversity or challenging situations, such as the transition from middle school to high school, which is a period marked by frequent adolescent issues (Blackwell et al., 2007). So, for other life-development stages or different time and space contexts during schooling, can the function of the growth mindset be manifested in relatively smooth life stages? To what extent can the growth mindset maximize its function in challenging environments with different characteristics? Whether its specificity can be highlighted deserves more attention. In a field survey with a wider scope and larger sample size, the association between a growth mindset and academic achievement was much weaker compared to the presentation of laboratory data (Bahník and Vranka, 2017), showing significant differences from the meta-analytical results of previous scholars such as Burnette. Although this does not imply that mindset does not have a positive effect on achieving goals, this study can be seen as a heterogeneous source. This also prompts us to reflect on whether the effectiveness of a growth mindset in predicting achievements in various fields is limited to specific research scenarios or small-scale samples. As research deepens and expands, investigators will face more complex environments and interaction mechanisms, and the exploration of mindset may be endless. The future direction of research efforts is to overcome these limitations in order to better explain the differences between existing and past research results.

### 5.5 Limitations

Firstly, in the initial phase of the study, a single Web of Science database was employed for the analysis of the literature. While other databases, including Scopus and Google Scholar, are also rich in literature, given the limited resources and time available, we prioritised focusing on WoS to achieve the best balance of research depth and efficiency. We acknowledge that this choice may bring certain limitations, especially that it may not cover all relevant literature. At the same time, the possibility of positive results bias cannot be ruled out.

Secondly, CiteSpace is a relatively straightforward visualisation tool. In future research, the combination of additional tools such as VOSviewer could facilitate a more in-depth and comprehensive analysis within this field. Furthermore, the utilisation of tools such as the Cochrane Risk of Bias Tool and the Critical Appraisal Skills Programme (CASP) could assist in the control of bias.

## 6 Conclusion

Based on 543 articles selected from the Social Sciences Citation Index database, this study used CiteSpace 6.2.R4 to evaluate and pinpoint the status, hot areas, and frontiers of growth-mindset. Researchers from Australia and countries/territories in North America, East Asia, and Western Europe have maintained relatively tighter cooperation with each other. Carol S. Dweck, Jeni L. Burnette, David S. Yeager, and Mary Murphy have high publication volumes and close connections with each other. Angela Duckworth has acted as a bridge among many researchers. Highly co-cited literature has mainly focused on the impacts of mindset and intervention measures. In the past two decades, the literature on mindset research has plunged into numerous hot topics in terms of implicit theory, intelligence, motivation, beliefs, achievements, academic performance, students, transitions, and psychological intervention. Based on burst detection, the field of growth-mindset research shows the following trends: (1) future research must pay more attention to fidelity in intervention studies, conduct rigorous manipulation tests at the statistical level, and improve causal relationship models between growth mindset and other variables and (2) use a multidisciplinary perspective to provide a deeper explanation of the formation mechanism of the growth mindset. Finally, (3) the function mechanisms of the growth mindset in different cultural backgrounds should be strengthened.
